# miR-137 acts as a tumor suppressor in papillary thyroid carcinoma by targeting CXCL12

**DOI:** 10.3892/or.2021.8157

**Published:** 2021-07-26

**Authors:** Su Dong, Meishan Jin, Ye Li, Peiyou Ren, Jia Liu

Oncol Rep 35: 2151-2158, 2016; DOI: 10.3892/or.2016.4604

Following the publication of this paper, it was drawn to the Editors attention by a concerned reader that certain of the western blotting data shown in Figs. 4D and 5B were strikingly similar to data appearing in different form in other articles by different authors. Owing to the fact that the contentious data in the above article had already been published elsewhere prior to its submission to *Oncology Reports*, the Editor has decided that this paper should be retracted from the Journal. After having been in contact with the authors, they agreed with the decision to retract the paper. The Editor apologizes to the readership for any inconvenience caused.

